# Antimicrobial activity and cytotoxicity of triterpenes isolated from leaves of *Maytenus undata* (Celastraceae)

**DOI:** 10.1186/1472-6882-13-111

**Published:** 2013-05-20

**Authors:** Tsholofelo Abednego Mokoka, Lyndy Joy McGaw, Ladislaus Kakore Mdee, Victor Patrick Bagla, Ezekiel Olugbenga Iwalewa, Jacobus Nicolaas Eloff

**Affiliations:** 1 Department of Paraclinical Sciences, Phytomedicine Programme, Faculty of Veterinary Science, University of Pretoria, Private Bag X04, Onderstepoort, 0110, South Africa; 2 Present address: Department of Health, Medicines Evaluations and Research, Private Bag X828, Pretoria, 0001, South Africa; 3 Present address: Department of Pharmacy, University of Limpopo, Private Bag X1106, Sovenga, 0727, South Africa; 4 Present address: Department of Biochemistry, Microbiology and Biotechnology, University of Limpopo, Private Bag X1106, Sovenga, 0727, South Africa; 5 Present address: Department of Pharmacology, Faculty of Pharmacy, Obafemi Awolowo University, Ile-Ife, Nigeria

**Keywords:** *Maytenus undata*, Celastraceae, Antibacterial, Antifungal, Cytotoxicity, Haemagglutination assay

## Abstract

**Background:**

Plants of the genus *Maytenus* belong to the family Celastraceae and are widely used in folk medicine as anti-tumour, anti-asthmatic, analgesic, anti-inflammatory, antimicrobial and anti-ulcer agents, and as a treatment for stomach problems. The aim of this study was to isolate and identify active compounds with antifungal activity from *Maytenus undata* after a preliminary study highlighted promising activity in crude extracts.

**Methods:**

Sequential extracts of *M. undata* leaves prepared using hexane, dichloromethane (DCM), acetone and methanol (MeOH) were tested for activity against *Cryptococcus neoformans*, a fungal organism implicated in opportunistic infections. Bioassay-guided fractionation of the hexane extract using *C. neoformans* as test organism was carried out to isolate antifungal compounds. The cytotoxicity of compounds isolated in sufficient quantities was evaluated using a tetrazolium-based colorimetric cellular assay (MTT) and a haemagglutination assay (HA).

**Results:**

The hexane extract was most active with an MIC of 20 μg/ml against *C. neoformans*. The triterpene compounds friedelin (1), epifriedelanol (2), taraxerol (3), 3-oxo-11α-methoxyolean-12-ene-30-oic acid (4), 3-oxo-11α-hydroxyolean-12-ene-30-oic acid (5) and 3,11-dihydroxyolean-12-ene-30-oic acid (6) were isolated. Compound 6 was isolated for the first time from a plant species. The antimicrobial activity of compounds 1, 3, 5 and 6 was determined against a range of bacteria and fungi implicated in opportunistic and nosocomial infections. Compounds 5 and 6 were the most active against all the tested microorganisms with MIC values ranging between 24 and 63 μg/ml, except against *Staphylococcus aureus* which was relatively resistant. Compounds 1 and 3 had a low toxicity with an LC_50_ > 200 μg/ml towards Vero cells in the MTT assay. Compounds 5 and 6 were toxic with LC_50_ values of 6.03±0.02 and 2.98±0.01 μg/ml, respectively. Compounds 1 and 3 similarly were not toxic to the red blood cells (RBCs) but compounds 5 and 6 were toxic, showing HA titer values of 1.33 and 0.67 respectively.

**Conclusions:**

Compounds 5 and 6 were the most active but were also relatively cytotoxic to monkey kidney cells and red blood cells, while the other isolated compounds were less active and less cytotoxic.

## Background

The world is currently experiencing challenges of increased resistance development against available antimicrobials. The AIDS pandemic has also resulted in large numbers of immunocompromised patients susceptible to opportunistic bacterial and fungal infections. Toxicity of currently used antimicrobial drugs, such as amphotericin B which causes hepatotoxicity, is a limiting factor in their use. Additionally, the cost of effective antimicrobials plays a vital role in their availability, mainly in developing countries.

The family Celastraceae includes 98 genera with approximately 1264 species [1], and has a long history of use in traditional medicine [2]. These plants are widespread in tropical and subtropical regions including North Africa, South America and East Asia, particularly in China [3]. *Maytenus* species are either trees or shrubs growing to a height of 1 to 9 m [4]. They are widely used in folk medicine as anti-tumour, anti-asthmatic and anti-ulcer agents, and as treatments for stomach problems, as analgesics, anti-inflammatories and antimicrobials [5-8].

*Maytenus undata* (Thunb.) Blakelock, commonly known as kokoboom in Afrikaans, and koko-tree or South African holly, is a shrub or tree about 1.5 to 10 m high, and is widespread in tropical southern Africa and in south and south-western Arabia [9]. This plant is also called idohame, egqwabali, ikhukhuze, indabulovalo, or inqayi-elibomvu in Zulu [10] and occurs in forests, at forest margins, in ravine forest among boulders and also in open woodland and bushveld, often on termite mounds. Muhammad et al. [7] investigated the chemical constituents of this plant species and isolated 12-oleanene and 3, 4-seco-12-oleanene triterpene acids that had antibacterial activity against *Staphylococcus aureus*, methicillin-resistant *Staphylococcus aureus* and *Pseudomonas aeruginosa*, with MIC values ranging from 3.25 to 50 μg/ml. In a preliminary screening study, *Maytenus undata* extracts had promising antifungal activity against *Cryptococcus neoformans*, with average minimum inhibitory concentration (MIC) values of 0.09 mg/ml after 24 h incubation [11], leading to a recommendation for further studies on the antifungal activity of *M. undata*. Owing to these results and the use of the species for antimicrobial purposes, the objective of the present study was to isolate antifungal compounds from *M. undata*, and to evaluate their antimicrobial activity and cytotoxicity. Specifically the bioassay-guided fractionation technique was employed to isolate the compounds, using *Cryptococcus neoformans* as the test organism against which activity was determined using bioautography and a broth microdilution assay. The purified compounds were then tested for efficacy against several other bacterial and fungal species, and for cytotoxicity against Vero African green monkey kidney cells and against equine red blood cells in a haemagglutination assay. Other compounds inactive against *C. neoformans* present in a high concentration were also isolated in the process of fractionation of the *M. undata* extract, and these were identified and also tested for activity against the suite of bacteria and fungi.

## Methods

### Plant collection and extraction

The leaves of *Maytenus undata* were collected from the Lowveld National Botanical Gardens, Nelspruit, South Africa in November 2005, just prior to conduction of the study. The identity of the plant material was confirmed by Prof JN Eloff. A voucher specimen was deposited at the HGWJ Schweickerdt Herbarium (University of Pretoria) under the number PRU 115680. The dried *M. undata* leaves (600 g) were ground to a fine powder and sequentially extracted (6000 ml × 3) at room temperature overnight with hexane, dichloromethane, acetone and methanol, successively. The extracts were concentrated under reduced pressure yielding 4.09% (24.55 g), 2.54% (15.23 g), 0.75% (4.47 g) and 13.69% (82.16 g) (w/w), respectively. All the extracts were subjected to bioautography and serial microdilution assays to determine MIC values against *Cryptoccocus neoformans*.

### Bioassay-guided fractionation and isolation

The hexane fraction (22.0 g) was chromatographed on silica gel 60 (400 g; 37 cm × 5 cm column). The column was initially eluted with dichloromethane (DCM, 200 ml), and then eluted with ethyl acetate:dichloromethane (EtOAc: DCM) mixtures of increasing polarity (EtOAc: DCM 1:9, 1:4, 3:7, 1:1, 4:1 and 1:0). The polarity of the eluting solvent was sequentially increased to MeOH: EtOAc (1:9, 1:4, 3:7, 1:1, 4:1 and 1:0). Thirteen fractions of 200 ml were collected, concentrated and analyzed by thin layer chromatography (TLC). Fractions containing similar constituents were combined. Fraction FH1 (2.1 g) obtained from elution with 100% DCM, was subjected to silica gel 60 (65.0 g) column chromatography (27 cm × 3 cm column). The column was eluted with a mixture of hexane:chloroform (CHCl_3_) (1:1) to yield compound 1. Fraction FH2 (4.5 g), obtained from elution with a mixture of EtOAc: DCM (1:9), was also chromatographed on silica gel 60 (160.0 g; 27.0 cm × 4.5 cm column) using EtOAc:hexane (1:9) to yield compounds 2 and 3. Fraction FHB (5.48 g) made up of the combination of fractions FH5 (EtOAc: DCM; 1:1), FH6 (EtOAc: DCM; 4:1) and FH7 (EtOAc: DCM; 1:0) was also separated by repeated silica gel 60 chromatography. Compound 4 was obtained by eluting the silica gel 60 column with a mixture of EtOAc:hexane (1:1) and compound 5 was obtained by eluting the column with MeOH: CHCl_3_ (5:95). Fraction FH8 (1.7 g) obtained from elution with a mixture of MeOH: EtOAc (1:9), was also repeatedly subjected to silica gel 60 column chromatography (50 g; 100cm × 1 cm column) using MeOH: CHCl_3_ (95:5) to yield compound 6.

### TLC fingerprinting

To facilitate dereplication, or the identification of known compounds in subsequent work, aliquots (10 μl) of 1 mg/ml solutions in chloroform (CHCl_3_) (equivalent to 10 μg) of each isolated compound were loaded on each of three aluminium-backed thin layer chromatography (TLC) plates (Silica gel 60 F_254,_ Merck) and eluted in three mobile systems of differing polarity [12]. The TLC systems used were as follows:

Benzene:ethanol:ammonium hydroxide (18:2:0.2) (BEA, non-polar)

Chloroform:ethyl acetate:formic acid (10:9:2) (CEF, intermediate polarity)

Ethyl acetate:methanol:water (EMW, 10:1.35:1) (polar)

The developed TLC plates were visualized under UV light at 254 and 365 nm to detect UV active or absorbing plant constituents. The plates were then sprayed with vanillin spray reagent constituting 0.1 g vanillin dissolved in 28 ml methanol, with the addition of 1 ml sulphuric acid [13] and heated at 110°C to optimal colour development.

### Bacterial and fungal cultures

The test bacteria comprised two Gram-positive species (*Staphylococcus aureus*, ATCC 29213 and *Enterococcus faecalis*, ATCC 29212) and two Gram-negative species (*Escherichia coli,* ATCC 25922 and *Pseudomonas aeruginosa,* ATCC 27853). The test fungi comprised *Candida albicans* and *Cryptococcus neoformans*, clinical isolates from the collection of the Department of Veterinary Tropical Diseases, University of Pretoria. Bacteria were maintained on Müller-Hinton (MH) agar at 4°C and were cultured in MH broth at 37°C. Fungi were maintained on Sabouraud Dextrose (SD) agar at 4°C and were inoculated in SD broth at 35°C and incubated overnight prior to conducting bioautography and microdilution assays.

### Bioautographic assays

Aliquots (10 μg) of each isolated compound were loaded on five aluminium-backed thin layer chromatography (TLC) plates (Merck, silica gel 60 F_254_) and were prepared in the solvent systems mentioned above. The plates were left uncovered in a dark place for several days to allow the eluting solvent to evaporate completely from the plates before being sprayed with a day-old actively growing suspension of bacterial and fungal cultures*.* The TLC plates were then incubated for 24 hours at 37°C under 100% relative humidity to allow the microorganism to grow on the plates. After overnight incubation the bioautograms were sprayed with 2 mg/ml *p*-iodonitrotetrazolium violet (INT, Sigma) in water and incubated for 30 minutes for the development of clear zones against a red background indicating inhibition of fungal growth by isolated bioactive compounds [14,15].

### Microdilution assay

The two-fold serial dilution microplate method [16] was used to determine the MIC values of plant extracts. This method has been used to evaluate antibacterial activities of plant extracts [16,17]. The method has been modified for evaluating antifungal activity [18]. Briefly, aliquots (100 μl) of 1 mg/ml solutions dissolved in acetone of the isolated compounds were serially diluted with distilled water in 96-well microtitre plates. A 100 μl aliquot of bacterial and fungal suspension was added to each well. Acetone was used as a solvent control and distilled water was used as a negative control because it is the least toxic to fungi of water-miscible extractants that would dissolve more non-polar compounds [19]. Amphotericin B and gentamicin were used as positive controls against fungi and bacteria respectively. For the antifungal assay, 40 μl of 0.2 mg/ml of INT was added to each well and the covered and sealed microtitre plates were incubated at 35°C overnight to ensure adequate colour development. For the antibacterial assay, INT was added after overnight incubation of the plant extracts with bacterial cultures at 37°C and incubated for a further 30 min to 1 hour until optimal colour development. The colourless tetrazolium salt acts as an electron acceptor and is reduced to a formazan product by biologically active organisms. Tests were carried out in triplicate and each experiment was repeated three times. The MIC was recorded as the lowest concentration of the extract that inhibited fungal or bacterial growth [16].

### Cytotoxicity

#### Tetrazolium-based colorimetric assay (MTT)

The MTT assay procedure [20] with slight modifications [21] was used to investigate cytotoxicity of the four isolated compounds 1, 3, 5 and 6 that were available in sufficient quantity to perform the test. Isolated compounds were tested for cytotoxicity against Vero monkey kidney cells obtained from the Department of Veterinary Tropical Diseases (University of Pretoria). The cells were maintained in minimal essential medium (MEM, Highveld Biological, Johannesburg, South Africa) supplemented with 0.1% gentamicin (Virbac) and 5% foetal calf serum (Adcock-Ingram). Cell suspensions were prepared from confluent monolayer cultures and plated at a density of 2 × 10^3^ cells into each well of a 96-well microtitre plate. Plates were incubated overnight at 37°C in a 5% CO_2_ incubator to allow attachment of cells prior to use in the cytotoxicity assay. Stock solutions of the isolated compounds (20 mg/ml) were prepared by dissolving them in DMSO. Appropriate dilutions of the isolated compounds were prepared in growth medium and added to the cells.

The viable cell growth after incubation for 120 hours with isolated compounds was determined using the 3-(4,5-dimethylthiazol)-2,5-diphenyl tetrazolium bromide (MTT) assay, a tetrazolium-based colorimetric assay [20]. After incubation, a washing step was included to remove traces of the test compounds, as it has been demonstrated that some botanical extracts or components can reduce MTT in the absence of living cells [22]. The supernatant was aspirated from the cells and 200 μl of phosphate buffered solution (PBS) used to rinse each well. The PBS was in turn aspirated and replaced with 200 μl of fresh growth medium. Following the washing step, 30 μl of MTT (5 mg/ml in PBS) was added to each well and the plates were incubated for a further 4 hours. The medium was aspirated from the wells and 50 μl DMSO added to each well to solubilize the formazan produced by mitochondrial activity. The absorbance was measured on a Versamax microplate reader (Molecular Devices) at 570 nm. Berberine chloride (Sigma) was used as a positive control and appropriate negative controls were included. The intensity of colour is directly proportional to the number of surviving cells. Tests were carried out in quadruplicate and each experiment was repeated three times. Selectivity index values were calculated by dividing cytotoxicity LC_50_ values by the MIC values (SI = LC_50_/MIC).

#### Haemagglutination assay (HA)

The haemagglutination assay was used to determine toxicity of the isolated compounds to erythrocyte membranes. The degree of toxicity is indicated by agglutination of the RBCs due to the change in the physiology of the blood cell membrane [23]. Equine erythrocytes fixed with formalin were prepared according to the technique of Sadique et al. [24]. Fresh blood was collected from a horse (Equine Research Centre, Faculty of Veterinary Sciences, University of Pretoria) into clean, dry glass tubes containing 3.8% sodium citrate (9 parts blood: 1 part 3.8% sodium citrate). The blood was centrifuged at 4000 rpm for 10 min. The packed RBCs were washed with 10 mM phosphate-buffered saline (PBS), pH 7.2, until a clear supernatant was obtained. The washed and packed RBCs were suspended in 5% (v/v) formaldehyde in PBS (1:12.3 v:v). The mixture was left at room temperature for 24 hours. The final fixed RBC were washed and centrifuged with PBS three times [23].

The haemagglutination assay was then conducted [23,24]. PBS (100 μl) was placed into U-shaped 96-well microtitre plates. The first row was used as a control without extracts. Isolated compounds 1, 3, 5 and 6 (100 μl of 3 mg/ml solution in acetone) were added to the second row and were serially diluted two-fold down the column. Then, 50 μl of horse RBCs were added to all the wells and incubated at room temperature for 1 hour. The presence of buttons in the centre of the well indicated no agglutination i.e. RBC membranes are not disrupted, hence no toxicity of the extract or isolated compounds. Acetylsalicyclic acid was used as a positive control. The haemagglutination (HA) titre value of the extracts was calculated as the reciprocal of the last dilution concentration showing agglutination, and was calculated using the formula HA titre value = 1/concentration value. A high HA titre value indicates a high level of toxicity which could cause serious toxic effects, particularly on the RBC blood group [23]. The samples were tested in triplicate in each assay and experiments were repeated three times.

## Results and discussion

### Bioactivity of extracts

The hexane fraction (MIC = 0.02 mg/ml) had a higher antifungal activity against *C. neoformans* than the other fractions, namely DCM (MIC = 0.04 mg/ml), acetone (MIC = 0.08 mg/ml) and MeOH (MIC = 0.56 mg/ml). It is clear that there was a good correlation between non-polarity and antifungal activity as the more non-polar the solvent used to prepare the fraction, the higher the activity. The hexane extract yielded a clear broad band on bioautograms against *C. neoformans* (results not shown). This implies that several active compounds that were not well separated were present. Hence, isolation of the active compounds was continued using the hexane extract.

### Chemical structures of the isolated compounds

**Figure 1:**
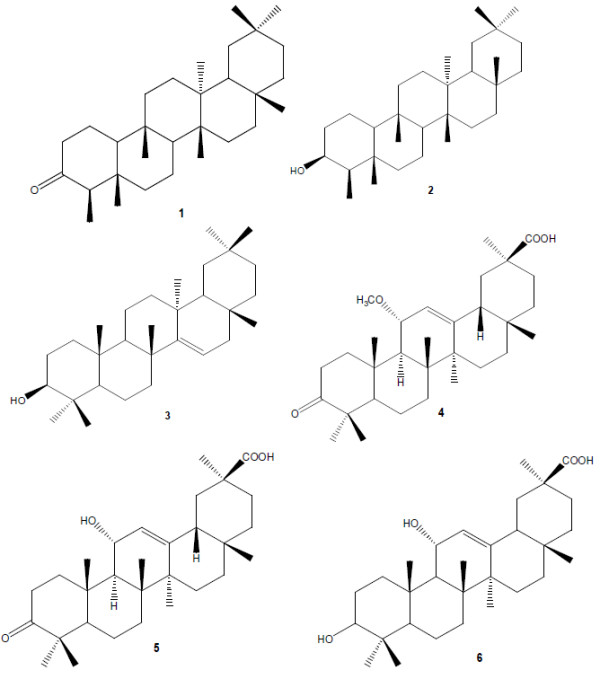
**Structures of compounds isolated from *Maytenus undata *leaves: friedelin (1), friedelan-3β-ol (2), taraxerol (3), 3-oxo-11α-methoxyolean-12-ene-30-oic acid (4), 3-oxo-11α-hydroxyolean-12-ene-30-oic acid (5), 3,11-dihydroxyolean-12-ene-30-oic acid (6).**

The structures of the isolated compounds 1–6 were determined by ^1^H and ^13^C NMR spectroscopy and by comparison of the spectral data with published data. The isolated compounds were identified as friedelin (1) (175 mg; 0.029%, w/w of hexane fraction) [25], friedelan-3β-ol (2) (15 mg; 0.0025%, w/w) [26], taraxerol (3) (155 mg; 0.026%, w/w) [27], 3-oxo-11α-methoxyolean-12-ene-30-oic acid (4) (23 mg; 0.0038%, w/w) [7], 3-oxo-11α-hydroxyolean-12-ene-30-oic acid (5) (82 mg; 0.014%, w/w) [7] and 3,11-dihydroxyolean-12-ene-30-oic acid (6) (68 mg; 0.011%, w/w). Compound 6 is closely related to glycyrrhetinic acid which is produced by the hydrolysis of glycyrrhizinic acid, a major component of *Glycyrrhiza glabra*[28]. Glycyrrhetinic acid has a keto group at C-11, while compound 6 has a hydroxyl group. This is the first report to our knowledge of the isolation and structure of compound 6 from a plant species. The structures of the isolated compounds are shown in Figure 1.

As an aid to dereplication, which prevents isolation of the same antifungal compounds from other plant species in future, the retention factor (R_f_) values for active compounds in three TLC solvent systems were determined. The R_f_ values of the isolated compounds ranged from 0.95 to 0.03 in BEA, 0.95 to 0.78 in CEF and 0.93 to 0.83 in EMW (Table 1).

**Table 1:** **Retention factor (R**_**f**_**) values of the isolated compounds in different mobile systems: friedelin (1), friedelan-3β-ol (2), taraxerol (3), 3-oxo-11α-methoxyolean-12-ene-30-oic acid (4), 3-oxo-11α-hydroxyolean-12-ene-30-oic acid (5), 3,11-dihydroxyolean-12-ene-30-oic acid (6)**

**Mobile phase**	**R**_**f**_**values of the isolated compounds**					
	**1**	**2**	**3**	**4**	**5**	**6**
BEA	0.95	0.85	0.73	0.14	0.04	0.03
CEF	0.95		0.91		0.77	0.78
EMW	0.93		0.92		0.87	0.83

### Bioautographic assays of the isolated compounds

Friedelin (1) did not produce any bands of inhibition against any of the tested microorganisms, meaning that no tested microorganisms were susceptible to the 10 μg of friedelin loaded on the TLC plate. Another explanation is that this non-polar compound may be so volatile that it may have evaporated from the chromatogram while the eluent was removed. Taraxerol (3) did produce clear bands against both *C. neoformans* and *E. coli*; however there were no clear growth inhibitory bands on the bioautograms sprayed with *P. aeruginosa* and *S. aureus*. All the microorganisms were sensitive to 3-oxo-11α-hydroxyolean-12-ene-30-oic acid (5) and 3,11-dihydroxyolean-12-ene-30-oic acid (6). Friedelan-3β-ol (2) and 3-oxo-11α-methoxyolean-12-ene-30-oic acid (4) were not isolated in sufficient quantities for bioautographic analysis.

### Minimum inhibitory concentrations

No microorganism was susceptible to friedelin (1) at the highest concentration tested (250 μg/ml) indicating that the lack of active bands in bioautography was not caused by the evaporation of friedelin. Only *E. faecalis* (MIC = 130±0.04 μg/ml) had some sensitivity to taraxerol (3). Both fungal species tested were sensitive to 3-oxo-11α-hydroxyolean-12-ene-30-oic acid (5) and 3,11-dihydroxyolean-12-ene-30-oic acid (6). However, 3-oxo-11α-hydroxyolean-12-ene-30-oic acid (5) was slightly more active against *Cryptococcus neoformans* (47±0.01 μg/ml) than *Candida albicans* (63±0.02 μg/ml) (Table 2). In general there was a good correlation between the bioautography and serial microdilution results.

**Table 2:** **MIC values and cytotoxicity of the hexane crude extract and isolated compounds from*M. undata***

	**Minimum inhibitory concentration (MIC, μg/ml)**						**Cytotoxicity**	
	***C. albicans***	***C. neoformans***	***E. coli***	***E. faecalis***	***S. aureus***	***P. aeruginosa***	**Vero cells (LC**_**50**_**)**	**HA titre value**
							**(μg/ml)**	
**Hexane extract**	300±0.01	20±0.00	120±0.06	80±0.03	630±0.00	300±0.00	76±0.05	1.60
**Compound 1**^1^	>250±0.00	>250±0.00	>250±0.00	>250±0.00	>250±0.00	>250±0.00	>200±0.00	**^2^
**Compound 3**	>250±0.00	>250±0.00	>250±0.00	130±0.04	>250±0.00	>250±0.01	>200±0.00	**^2^
**Compound 5**	63±0.02	47±0.01	32±0.02	32±0.05	>250±0.01	36±0.04	6.03±0.02	1.33
**Compound 6**	63±0.05	47±0.02	24±0.05	63±0.04	>250±0.00	32±0.02	2.98±0.01	0.67
**Amphotericin B**	0.16±0.00	0.16±0.00						
**Gentamicin**			6.30±0.00	25±0.00	15±0.01	3.20±0.03		
**Berberine**							12.35±0.00	
**Acetylsalicyclic acid**								0.80

^1^friedelin (**1**), friedelan-3β-ol (**2**), taraxerol (**3**), 3-oxo-11α-methoxyolean-12-ene-30-oic acid (**4**), 3-oxo-11α-hydroxyolean-12-ene-30-oic acid (**5**), 3,11-dihydroxyolean-12-ene-30-oic acid (**6**).

^2^** Low haemagglutination (HA) titre value (compounds not toxic).

A review of the available literature indicates that not one of the isolated compounds has been tested for antifungal activity against *C. neoformans*, but some of the compounds have been tested against *C. albicans* and some bacterial species. Friedelin (1) has antifungal activity against *C. albicans* with an MIC value of 2.44 μg/ml [29]. Our results could not confirm the antifungal activity of this compound against *C. albicans*. In addition, the antibacterial activity of friedelin (1) against *E. faecalis* with an MIC value of 0.61 μg/ml was reported [29]. No antibacterial activity against *P. aeruginosa* and *S. aureus* was detected [29], in agreement with our results.

*Staphylococcus aureus* was least sensitive to all the tested compounds, with MIC values greater than 250 μg/ml, the highest concentration tested. All the other pathogens were sensitive to 3-oxo-11α-hydroxyolean-12-ene-30-oic acid (5) with MIC values of 36±0.04 μg/ml and 32±0.02 μg/ml for *P. aeruginosa* and *E. coli* respectively. Muhammad and colleagues [7] showed that compound 5 was active against *S. aureus* and *P. aeruginosa* with MIC values >10 μg/ml and 6.25 μg/ml, respectively.

Compound 6 (11-dihydroxyolean-12-ene-30-oic acid) has a similar structure to glycyrrhetinic acid and it was more active against *E. coli* (MIC = 24±0.05 μg/ml) than any of the other microorganisms tested. Glycyrrhetinic acid did not inhibit the growth of *E. coli* (KCTC 1682) and *C. albicans* (KCTC 7270) [30], but compound 6 had substantial activity against most of the tested microorganisms in this study with MIC values ranging from 24 to 63 μg/ml, except against *S. aureus* which was less susceptible. The reason for this difference in activity might be due to the presence of two hydroxyl groups in compound 6, compared to glycyrrhetinic acid which contains a keto group and lacks a hydroxyl group.

Compounds 1 and 3 were not highly active against the tested microorganisms, especially against *C. neoformans.* Amphotericin B had an MIC value of 0.16±0.00 μg/ml but both compounds 5 and 6 had MIC values of 47 μg/ml against *C. neoformans*. The crude hexane extract had an MIC of 20±0.00 μg/ml against *C. neoformans*, and was therefore more active than compounds 5 and 6. In addition, hexane crude extract bioautograms against *C. neoformans* displayed a broad clear zone of fungal growth inhibition, indicating the presence of antifungal constituents with very similar retention indices. Hence, the separation of activity into single pure components was not achieved, resulting in a reduced antifungal activity. This suggests the possibility of synergistic effects between active or even non-active plant constituents in the *M. undata* extract which might influence the uptake of active constituents by microorganisms. This phenomenon is possible in plant extracts containing numerous constituents responsible for different properties within the plant. Some of the compounds which showed activity on bioautograms were not isolated in this study because they were present in quantities too low to isolate using these methods. These compounds may contribute to synergistic effects, resulting in total activity of the crude extract.

### Cytotoxicity

#### MTT assay

Both friedelin (1) and taraxerol (3) were slightly toxic to the Vero cells with an LC_50_ higher than the highest concentration (200 μg/ml) tested. The compounds 3-oxo-11α-hydroxyolean-12-ene-30-oic acid (5) and 3,11-dihydroxyolean-12-ene-30-oic acid (6) were relatively toxic with LC_50_ values of 6.03±0.02 and 2.98±0.01 μg/ml, respectively. Berberine had an LC_50_ of 12.4±0.00 μg/ml, calculated from the regression curve. Upon calculating the therapeutic index of compounds 5 and 6 against five of the pathogens by dividing the LC_50_ by the MIC, values between 0.05 and 0.12 were obtained, indicating that the compounds are much more toxic to the Vero cells than to the pathogens. Selectivity index (SI) values calculated using the cytotoxicity results against Vero cells and MIC values (Table 3) showed that the crude hexane extract had the best SI value of 3.80 against *C. neoformans*, but none of the compounds had SI values above 1, indicating that bioactivity was most likely owing to general toxic effects of the plant constituents isolated. It is possible that structure-activity studies and chemical modification experiments could reduce toxicity and enhance activity of the isolated compounds to increase their potential usefulness in future.

**Table 3:** **Selectivity index values of the hexane crude extract and isolated compounds from *M. undata***

	**Selectivity index values (LC**_**50**_**/MIC)**					
	***C. albicans***	***C. neoformans***	***E. coli***	***E. faecalis***	***S. aureus***	***P. aeruginosa***
**Hexane extract**	0.25	3.80	0.63	0.95	0.12	0.25
**Compound 1**^1^	NC^2^	NC	NC	NC	NC	NC
**Compound 3**	NC	NC	NC	NC	NC	NC
**Compound 5**	0.096	0.13	0.19	0.19	NC	0.17
**Compound 6**	0.047	0.063	0.12	0.047	NC	0.093

^1^ friedelin (**1**), friedelan-3β-ol (**2**), taraxerol (**3**), 3-oxo-11α-methoxyolean-12-ene-30-oic acid (**4**), 3-oxo-11α-hydroxyolean-12-ene-30-oic acid (**5**), 3,11-dihydroxyolean-12-ene-30-oic acid (**6**).

^2^NC = not calculated.

#### Haemagglutination assay

As with the Vero cell assay friedelin (1) and taraxerol (3) were not toxic to the formaldehyde-fixed red blood cells even at the highest concentration tested (3000 μg/ml). However, compounds 5 and 6 caused agglutination of the RBCs. These compounds were toxic with high HA titre values of 1.33 and 0.67 respectively to the formaldehyde-fixed RBCs, so compound 5 was more toxic to the RBCs than compound 6. The positive control, acetylsalicyclic acid, showed less toxicity than compound 5 and no significant difference was observed when compared with compound 6.

### Chemical structures and activity

The structures of the isolated compounds 3-oxo-11α-methoxyolean-12-ene-30-oic acid (4), 3-oxo-11α-hydroxyolean-12-ene-30-oic acid (5) and 3,11-dihydroxyolean-12-ene-30-oic acid (6) belong to the 12-oleanene group, just like oleanolic acid and its derivatives such as ursolic acid and betulinic acid with known antifungal and antibacterial activity [31,32]. From the current literature it is evident that a simple structural modification of a parent compound can dramatically influence biological activity [33,34]. The biological activity can either increase or decrease depending on the modification that has occurred. Compounds with additional oxygen function(s) to those in oleanolic acid and pomolic acid showed decreased anti-HIV activity [33,34]. This means that active compounds with fewer polar oxygen substituents may inhibit microbial growth because of the high hydrophobic interaction with target microbial cells. The compounds 3-oxo-11α-hydroxyolean-12-ene-30-oic acid (5) and 3,11-dihydroxyolean-12-ene-30-oic acid (6) isolated in this study can potentially act as lead compounds in the development of more potent antimicrobials against bacterial and fungal infections.

## Conclusions

This study investigated the potential significance of *Maytenus undata* extracts and isolated compounds in the development of new antibacterial and antifungal drugs, following the widespread use of *Maytenus* species in traditional medicine for antimicrobial purposes. In vitro antimicrobial activity evaluation of pure compounds from crude plant extracts can play a major role in the discovery of potential new drug leads. The simultaneous biological activity and cytotoxicity evaluation of isolated pure compounds and plant extracts provides valuable information regarding the prospective use of medicinal plants as sources of new drugs. Also, it emphasizes the rationale for using medicinal plants in folk medicine. The antibacterial and antifungal activity of *M. undata* extracts and isolated compounds may validate the traditional use of the plant as an antimicrobial agent and as a treatment for stomach problems.

With many people relying on plant products for their primary health care needs, possible toxicity to humans is a worrying factor. Therefore, it is useful to highlight where general toxicity is not responsible for the observed biological activity. In this study, the most active isolated compounds, 3-oxo-11α-hydroxyolean-12-ene-30-oic acid (5) and 3,11-dihydroxyolean-12-ene-30-oic acid (6) were relatively cytotoxic to monkey kidney cells. Structural modifications of active compounds may play a crucial role in advancing their development into useful drugs with reduced toxicity. The discovery of active compounds against opportunistic infections like *C. albicans*, *C. neoformans* and *S. aureus* will add significant value in the treatment of various human ailments such as HIV-AIDS with its associated opportunistic pathogens and sexually transmitted infections (STIs). Therefore, more directed research is needed to explore the ability of plants to enhance the discovery and development of new chemical entities.

## Abbreviations

DCM: Dichloromethane; EtOAc: Ethyl acetate; HA: Haemagglutination assay; INT: *p*-iodonitrotetrazolium violet; MeOH: Methanol; MH: Müller-Hinton; MIC: Minimum inhibitory concentration; MTT: 3-(4,5-dimethylthiazol-2-yl)-2,5-diphenyltetrazolium bromide; RBCs: Red blood cells; SD: Sabouraud Dextrose; TLC: Thin Layer Chromatography.

## Competing interests

The authors declare that they have no competing interests.

## Authors’ contributions

TAM conducted all the practical work and drafted the manuscript, LJM co-supervised the research design and implementation, was instrumental in performing the cytotoxicity study and edited the manuscript, LKM assisted with the isolation and structure elucidation of the compounds, VPB assisted with various aspects of the practical work, EOI helped with the haemagglutination study and JNE supervised the design of the research and helped edit the manuscript. All authors read and approved the final manuscript.

## Pre-publication history

The pre-publication history for this paper can be accessed here:

http://www.biomedcentral.com/1472-6882/13/111/prepub
